# Endometriosis-associated recto-sigmoid cancer: a case report

**DOI:** 10.1186/s12885-018-4797-4

**Published:** 2018-09-20

**Authors:** Ningning Li, Weixun Zhou, Lin Zhao, Jiaolin Zhou

**Affiliations:** 1 Department of Medical Oncology, Peking Union Medical College Hospital, Peking Union Medical College and Chinese Academy of Medical Sciences, Beijing, 100730 China; 2 Department of Pathology, Peking Union Medical College Hospital, Peking Union Medical College and Chinese Academy of Medical Sciences, Beijing, 100730 China; 3 Department of General Surgery, Peking Union Medical College Hospital, Peking Union Medical College and Chinese Academy of Medical Sciences, Beijing, 100730 China

**Keywords:** Endometriosis-associated carcinoma, Bowel endometriosis, Malignant degeneration

## Abstract

**Background:**

Endometriosis is a relatively common condition in women of reproductive age. Malignant transformation of intestinal endometriosis is a very rare event. We report a case in which a patient with a history of endometriosis underwent surgery for malignant intestinal endometriosis.

**Case presentation:**

A 55-year-old woman complained of rectorrhagia and intermittent abdominal pain. A neoplasm was revealed by colonoscopy, CT scan and F18-FDG PET/CT of the recto-sigmoidal colon. The patient underwent a rectal anterior resection, hysterectomy and bilateral salpingo-oophorectomy for treatment. According to the histological and immunohistochemical presentation, the diagnosis of endometriosis-associated recto-sigmoid cancer was confirmed. The patient was treated with adjuvant chemotherapy for 6 months. During the follow-up appointment 22 months later, there was clinical and radiographic evidence of recurrence in the rectum. The patient received chemotherapy again and will receive another surgery after two more cycles of chemotherapy.

**Conclusion:**

We report a case of malignant intestinal endometriosis. Although there is no standard therapy for malignant intestinal endometriosis due to the rarity of this disease, surgery and adjuvant chemotherapy seemed to be rational. This case indicates that local recurrence may be a common situation after standard therapy.

## Background

Endometriosis is a common proliferative disease among women. It occurs in 4–17% of women of reproductive age. It refers to the occurrence of the endometrial tissue growing outside of the uterus. Many organs can be involved in this process, including the fallopian tubes, ovaries, vagina, cervix and the Pouch of Douglas; extragonadal sites include the gastrointestinal tract, bladder, lungs, central nervous system, and even the skin [1]. Endometriosis with intestinal involvement is rare. The most common location in the intestinal tract is the sigmoid colon and the rectum (50–90%) [2, 3]. The first case of malignant transformation was described in 1925. While malignant transformation of ovarian endometriosis is well-known, extraovarian endometriosis associated with cancer is rare, and therapeutic management is not standardized. We report a case of a female patient with a recto-sigmoidal lesion that was initially diagnosed as a primary intestinal cancer. Pathology confirmed the diagnosis of an endometrial carcinoma derived from endometrial tissue in the intestinal wall. The patient had a history of prior abdominal surgery for an endometriotic ovarian cyst.

## Case Presentation

### Patient information

A 55-year-old woman was referred to the Peking Union Medical College Hospital in September 2015 for rectorrhagia and intermittent abdominal pain lasting 6 months. The patient had a past medical history of ovarian endometriosis and had undergone excision of bilateral ovarian chocolate cysts in 1988 when she was 30 years old. Histological examination showed benign bilateral endometriosis of the ovaries. The patient was not treated with any hormonal therapy following hysterectomy. She has a familial history of endometriosis comprising her mother, one sister and one aunt.

### Clinical findings

Physical examination of the patient was unremarkable, except for the tenderness of the left lower quadrant of the abdomen.

### Diagnostic assessment

**Fig. 1:**
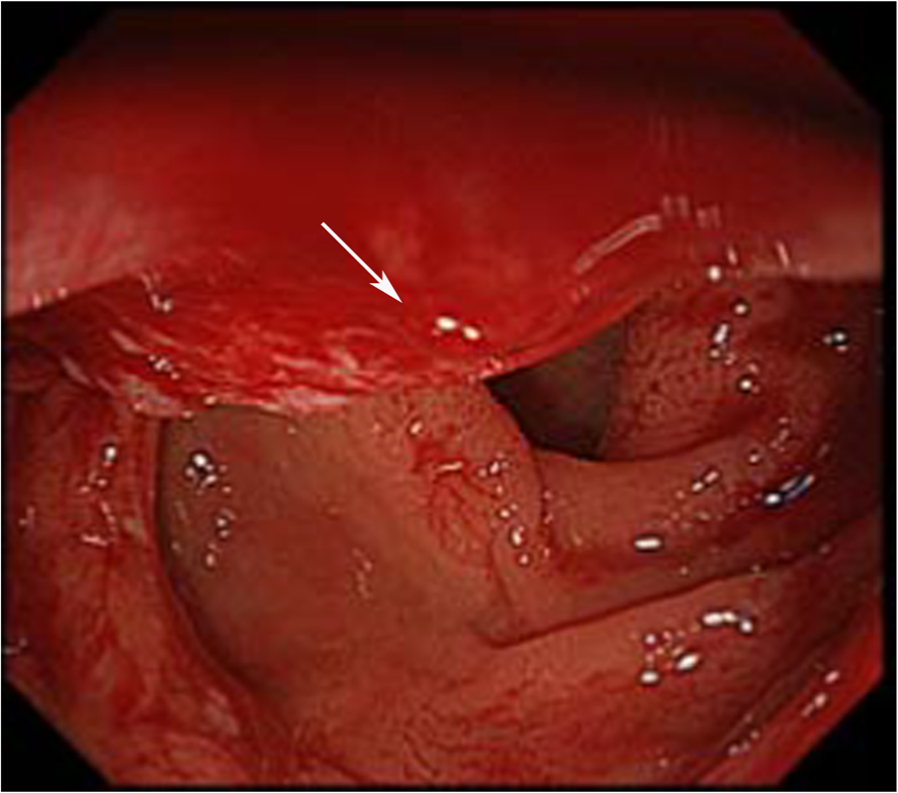
An endoscopic view of the recto-sigmoidal lesion

**Fig. 2:**
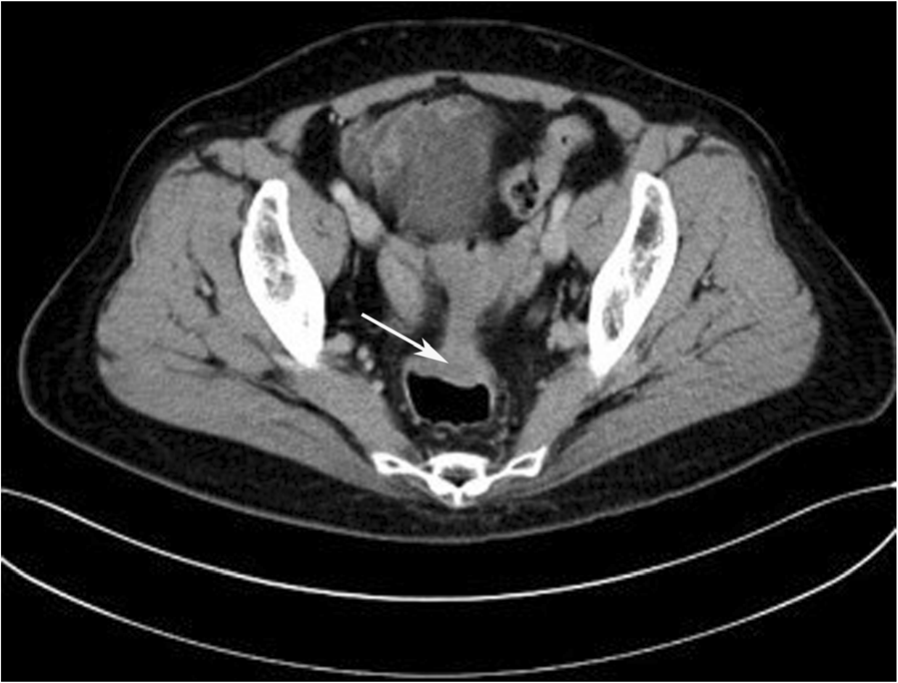
Contrast-enhanced computed tomography (CT) image showing diffuse and irregular thickening of the wall of the recto-sigmoidal junction

**Fig. 3:**
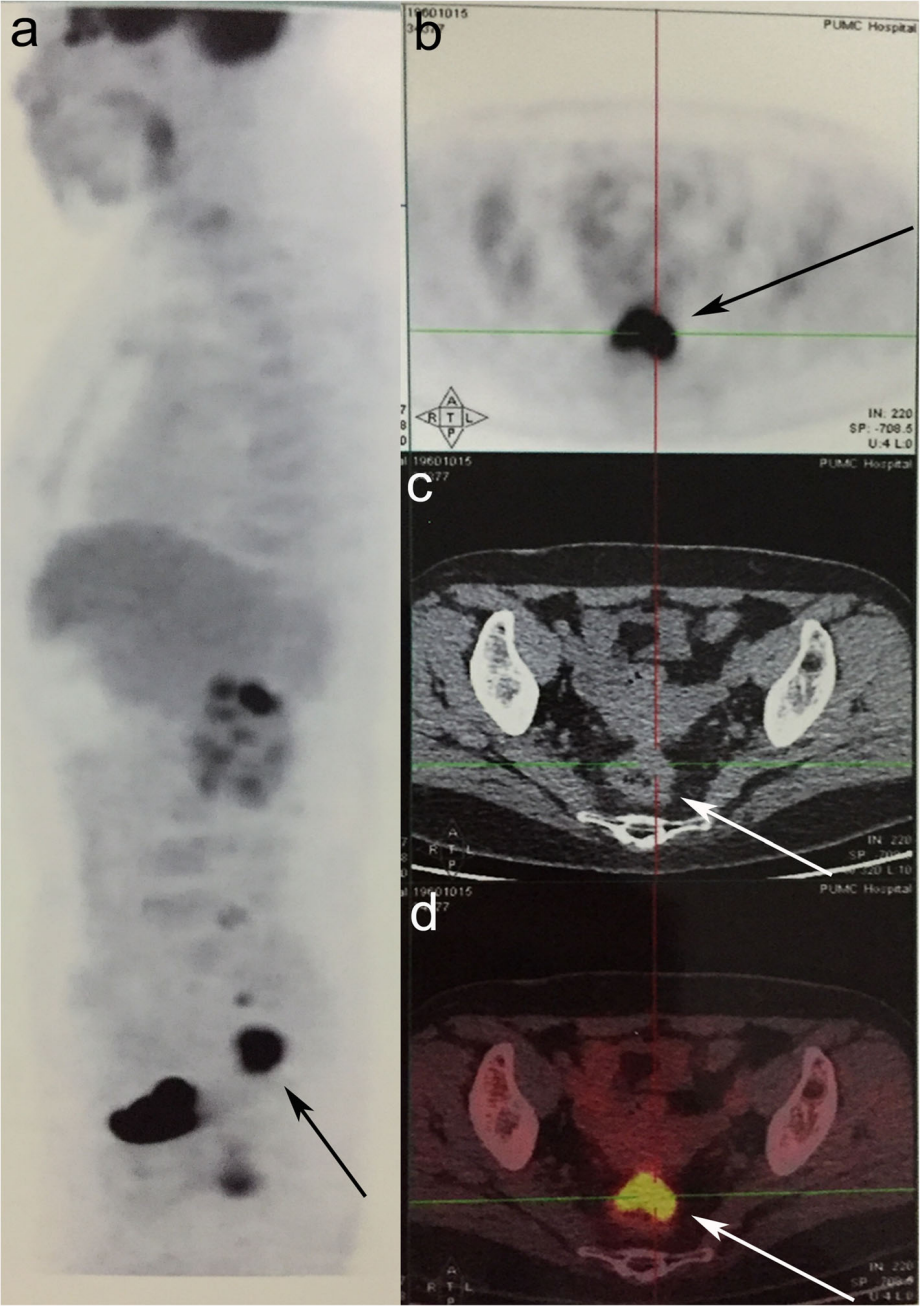
True positive focal-fluorodeoxyglucose (FDG) colonic activity in a positron emission tomography/computed tomography (PET/CT). **a** FDG-PET/CT maximum intensity projection image showing the intensity of a hypermetabolic wall thickening in the recto-sigmoid colon (maximum standardized uptake value of 15.7). **b** FDG images (**c**) CT images and (**d**) hybrid images of the malignancy

Serum CA125, CEA, and CA199 were within a normal range. A colonoscopy was performed (Fig. 1), which revealed an ulcerated fleshy neoplasm that was 15 cm from the anal margin and blocked 50% of the passage of the endoscope. It was possible to pass the endoscope beyond the lesion. The mass appeared to bleed relatively easily and had a diameter of 2 × 3 cm. The surface of the lesion was irregular, and the margin was unclear. The surrounding colonic mucosa was rough. The results of the scan also revealed a 0.8 cm sessile polyp in the ascending colon. Multiple endoscopic biopsies were taken. Pelvic ultrasound showed multiple uterine leiomyomas, which presented as multiple heterogeneous internal echoes. A computed tomography scan revealed eccentric thickening of the wall of the recto-sigmoidal junction (Fig. 2). A whole body FDG-positron emission tomography (PET) was requested (Fig. 3). F18-FDG PET/CT imaging showed local thickening and narrowing of the recto-sigmoid colon wall and hypermetabolic lesions. FDG-PET/CT also found enlarged pelvic lymph nodes with pathologic FDG-uptake. Since the pathology obtained from the colonoscopy showed evidence of a metastatic adenocarcinoma, and the lesion was confined to the pelvic cavity, laparoscopic surgery was performed on October 20, 2015. During the procedure, a 3.5-cm ulcerated mass was identified above the peritoneal reflection with multiple adhesions to the uterine wall. The surgeons examined the pelvic cavity carefully and found apparent adhesions in the pelvic cavity due to the previous surgical procedures. The adhesions present between the tumour and the uterus were severed. There were no obvious tumours on either of the ovaries. A rectal anterior resection with lymphadenectomy was performed. The lymph nodes around the common, internal, and external iliac arteries, the middle rectal artery root, and the obturator space were dissected. Hysterectomy and bilateral salpingo-oophorectomy were performed. There was no residual macroscopic tumour present at the completion of the surgery.

**Fig. 4:**
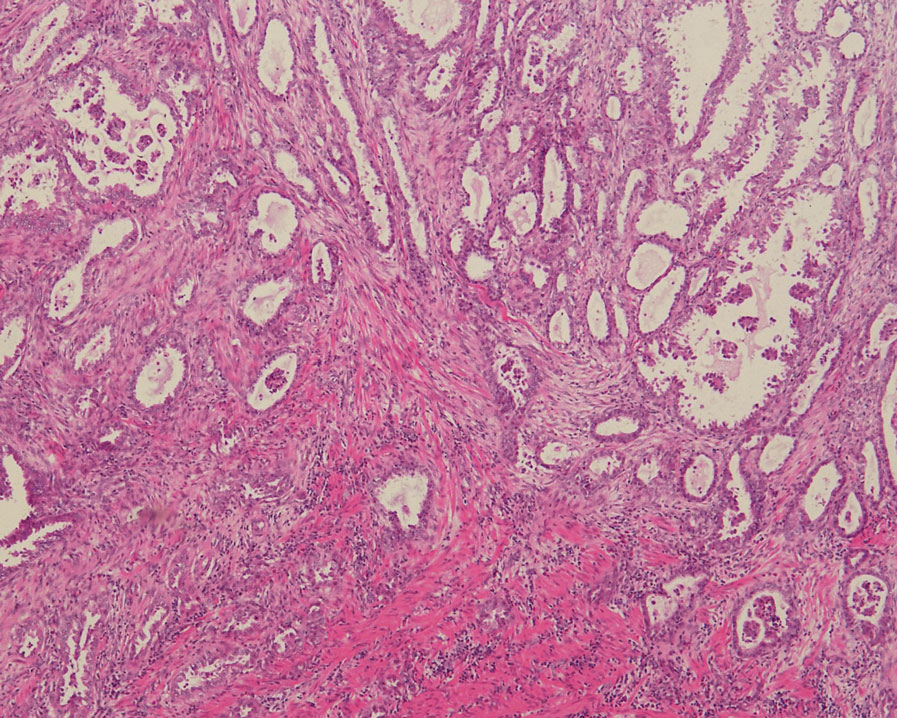
Adenocarcinoma infiltrating the colon. The tumour cells form irregular glands

**Fig. 5:**
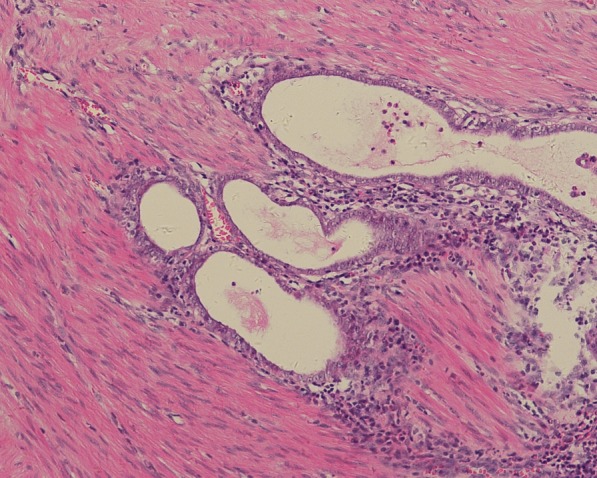
A small foci of endometriosis near the tumour in the muscularis propria of the colon

**Fig. 6:**
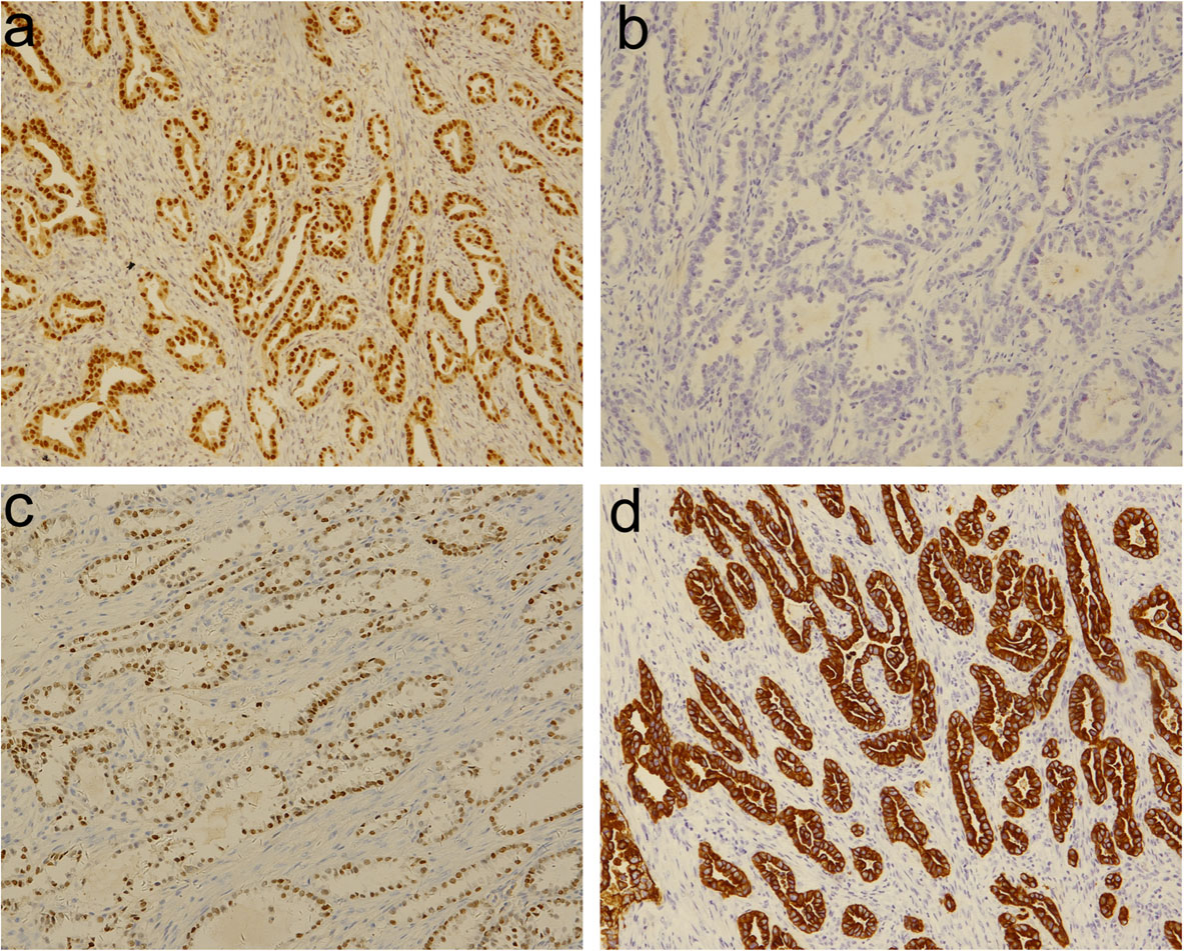
Immunohistochemstry of the tumour shows PAX-8 positive (**a**), CDX-2 negative (**b**), CK7 positive (**c**) and ER positive (**d**) cells

The endoscopic biopsy specimens were fixed in 10% buffered formalin and embedded in paraffin. Four μm sections were stained with standard haematoxylin and eosin (H&E). Immunohistochemical stains were performed using the following antibodies: CK7, CDX-2, CA-125, Villin (Dako); CK20 (OriGene Technologies); PAX-8, WT-1(XiYa Biology); P16, ER, PR (VENTANA); P53 (Maixin Biotech. Co., Ltd.). The H&E slides and immunohistochemical stains were reviewed by two pathologists. The excised bowel was sent for histological analysis. The pathologic specimen consisted of an ulcerated mass with grey, solid, friable cut surface. The mass measured 3.5 × 2 × 1.2 cm and was attached to the wall of the colon. The mass involved the muscularis propria macroscopically. Microscopic sections revealed a high-grade serous carcinoma extending through all of the layers of the intestine (Fig. 4). Tumour cells appeared as irregular tubular structures, moderately differentiated, with no sign of atypical adenomatous hyperplasia. The section margins were free of tumour, but the circumferential resection margin (CRM) was positive. Eight out of 30 lymph nodes that were collected were positive. The uterus and the ovaries were free of cancer. According to the preliminary pathological diagnosis, the TNM staging was determined to be pT4bN2bM0 (IIIC) based on the AJCC Staging System. The final histology was confirmed after discussion between several pathologists who diagnosed the patient with pronounced focal atypical endometriosis in the intestinal wall and the right ovary (Fig. 5). The immunohistochemical profile showed CK7 positivity and CK20 negativity, as well as negative CDX2 (Fig. 6). The case was positive for CA125, PAX8, P16 and mildly positive for oestrogen receptors. The Ki67 index was 30%. Based on the immunohistochemical characteristics of the primary intestinal adenocarcinoma, it was determined that the lesion was an endometrial carcinoma deriving from colorectal endometriosis.

### Therapeutic intervention

The patient received adjuvant chemotherapy consisting of 175 mg/m2 Taxol and AUC5 carboplatin for eight cycles. The patient experienced reversible chemotherapy-induced myelosuppression and gastrointestinal reactions during chemotherapy. The timeline of the process was shown in Table 1.

**Table 1:** Important dates in the case

Date	Event
September 2015	Doctor visit
October 20th, 2015	Laparoscopic surgery
November 19th to April 30th, 2016	Chemotherapy
December 2016	Recurrence
March 30th, 2017	Chemotherapy

### Follow-up and outcomes

At 23-months, the patient had a follow-up appointment and reported difficult defecation at the 22nd month. The CT scan, PET-CT and colonoscopy showed local recurrence in the lower rectum, which was confirmed by pathological diagnosis. The patient has received Taxol and carboplatin chemotherapy again and will receive another surgery after two cycles of chemotherapy.

## Discussion

Endometriosis is a common gynaecological disease, but malignant transformation of endometriosis is rare. The malignancies arising from ovarian endometriosis are most common, followed by those arising from foci in the pelvic peritoneum and the colorectal tract [1, 4, 5]. In a systematic review [6], 80% of all extragonadal endometriosis-associated neoplasms occurred in the rectum and sigmoid colon. Histologically, they were mostly adenocarcinomas. Adenocarcinoma arising from endometriosis often mimics primary intestinal adenocarcinoma. Pathological and immunohistochemical staining are essential to make an accurate clinical diagnosis. The histological diagnosis is based on Sampson’s criteria, which are the basic criteria for the diagnosis of endometriosis-associated malignancy [7]. Immunohistochemical examinations are also useful to distinguish between adenocarcinoma arising from endometriosis and primary intestinal adenocarcinoma. Endometrioid glands are usually immunoreactive for CK7, and ER, and stromal cells are positive for CD10 and ER. Intestinal glands express CDX2 and CK20, while displaying negative expression of CK7, ER or CD10. PAX8 was shown to be expressed in gynaecological cancers [8]. To date, there is no consensus on the therapeutic approach to treating endometriosis-associated malignancies. However, surgery with radical resection of the tumour should be performed if possible. The overall effectiveness of chemotherapy is unknown. There has been a broad consensus that the therapeutic principle and regimens applied to ovarian cancer also apply to endometriosis-associated ovarian cancer (EAOC) [9]. To this end, platinum-taxane combinations should be taken as the first choice for the standard adjuvant therapy [10]. To date, it is recommended that patients who are diagnosed with extragonadal endometriosis-associated carcinomas, which are confined to the lower pelvic cavity, may benefit from adjuvant pelvic irradiation [11]. Neoadjuvant preoperative chemotherapy and radiation therapy may be an appropriate additional measure. Considering the rarity of this disease, the application of neoadjuvant therapy must be further investigated. Additionally, the application of endocrine therapy in endometrial carcinoma should be investigated, as hormone-therapy may have a similar efficacy on gestagen receptor-positive endometriosis-associated malignancies. Our report has several limitations. First, we report just one case, and the individual characteristics cannot be generalized to other patients. Second, in the case presented, the patient did not receive adjuvant radiotherapy, which may decrease the local recurrence rate. Lastly, the short follow-up relative to the long-term prognosis must be verified. We will report the long-term follow-up and will pay close attention to similar cases.

## Conclusion

Endometriosis-associated intestinal cancer is rare, and it requires a clinical suspicion for diagnosis. In the case for patients with a medical history of endometriosis and / or suspected colorectal malignancy, immunohistochemical examinations are important for differential diagnosis. Currently, surgery, chemotherapy, radiotherapy and hormonal therapy are selected according to the particular situation of the tumour and the individual. The prognosis of these rare tumours may be improved with the application of targeted medication and immunotherapy.

## Abbreviations

AUC: Area under the curve
CA125: Cancer antigen 125
CA199: Carbohydrate antigen 19–9
CD10: Lymphocyte antigen 10
CDX2: Caudal-related homeodomain transcription 2
CEA: Carcinoembryonic antigen
CK20: Cytokeratin-20
CK7: Cytokeratin-7
CRM: Circumferential resection margin
CT: Computer Tomography
EAOC: Endometriosis-associated ovarian cancer
ER: Estrogen receptor
F18- FDG PET/CT: F18-fluorodeoxyglucose Positron Emission Tomography/Computed Tomography
H&E: Haematoxylin and eosin stain
Ki67: A nuclear protein that is associated with cellular proliferation
P16: Multiple tumour suppressor 1
P53: Cellular tumour antigen
PAX-8: Paired box gene 8
PR: Progesterone receptor
WT-1: Wilms tumour protein
